# A qualitative study to explore dietary knowledge, beliefs, and practices among pregnant women in a rural health zone in the Democratic Republic of Congo

**DOI:** 10.1186/s41043-022-00333-7

**Published:** 2022-11-22

**Authors:** Benito Kazenza Maykondo, Christiane Horwood, Lyn Haskins, Sphindile Mapumulo, Mala Ali Mapatano, Branly Mbunga Kilola, Marc Bosonkie Mokanisa, Anne Hatloy, Vaughn M. John, Paulin Mutombo Beya Wa Bitadi

**Affiliations:** 1 Department of Nutrition, Kinshasa School of Public Health, University of Kinshasa, Kinshasa, Democratic Republic of Congo; 2 Centre for Rural Health, University of KwaZulu Natal, Berea, South Africa; 3 School of Education, University of KwaZulu Natal, Berea, South Africa; 4 Fafo Institute for Labour and Social Research, Oslo, Norway

**Keywords:** Knowledge, Practice, Pregnant women, Food taboos, Diet, Nutrition, Democratic Republic of Congo

## Abstract

**Background:**

A nutritious and healthy diet during pregnancy is essential for the health of both mother and baby. Inadequate dietary intake during pregnancy contributes to maternal malnutrition and can have lifelong effects on the health of the child. Maternal malnutrition is common in many low-income countries, including the Democratic Republic of Congo (DRC). Kwango province, DRC, has a high prevalence of malnutrition among all population groups, including macro and micronutrient deficiencies among pregnant women. The study aimed to explore the dietary knowledge and practices of a pregnant woman in this area.

**Methods:**

This study adopted a qualitative approach using in-depth interviews (IDIs) with pregnant women and key informants, and focus group discussions (FGDs) with fathers and grandmothers in the community, to explore women’s knowledge and practice about diet during pregnancy. Data were collected between January and April 2018. IDIs were conducted with pregnant women who were recruited at antenatal clinics during their second and third trimesters. IDIs were undertaken with selected key informants, who were health workers providing care to pregnant women, and included doctors, nurses, nutritionists, and community health workers. All IDIs and FGDs were audio-recorded, transcribed verbatim, and translated to English. The triangulation method and thematic analyses were used.

**Results:**

Overall, women showed good general knowledge about nutrition and the need for increased and varied foods during pregnancy, but little technical knowledge about nutrients and sources of nutrition. Healthcare facilities, media, NGOs, and family members were the main sources of nutritional information. However, women were unable to put this knowledge into practice, primarily due to poverty and poor access to a variety of foods. The Popokabaka community accessed food from farming, fishing, and the market, although purchasing food was frequently unaffordable. Cassava flour was the most common daily food. Food taboos, traditional practices, and late ANC attendance were identified as factors that influenced dietary practices.

**Conclusions:**

Various social, economic, and environmental factors within the local community influenced dietary practices among pregnant women in rural DRC. A comprehensive approach is required to improve nutrition, and address food insecurity, cultural practices and improve the health outcomes of both mother and child.

## Introduction

Poor diet during pregnancy may have irreversible consequences, leading to inadequate maternal nutrition and adverse pregnancy outcomes for mother and child [1–3]. A good diet during pregnancy supports optimal maternal weight gain, protects against maternal anemia, reduces preterm delivery, and improves birthweight [4]. Moreover, healthy eating during pregnancy is essential to meet the increased energy and nutrient requirements of the mother and fetus. Thus, nutrient intake and weight gain during pregnancy are the two most important modifiable factors influencing maternal and fetal outcomes [5].

The World Health Organization (WHO) recommends that pregnant women increase their intake of energy, vitamins, and minerals through increased macronutrient and micronutrient consumption during pregnancy. Women are advised to eat a variety of food groups including green and orange vegetables, meat, fish, beans, nuts, whole grains, and fruit, and to increase their overall food intake during pregnancy [6]. Increasing the intake of nutrients is most important among women with a low pre-pregnancy body mass index (BMI).

The provision of nutrition education is a widely implemented strategy that has been shown to improve dietary knowledge and practices during pregnancy, and improve perinatal outcomes, particularly among women with pre-existing undernutrition [7]. A review of intervention studies showed that nutritional education to increase protein and energy intake decreased the risk of preterm birth and low birth weight, with a relative increase of almost half a kilogram in the birth weight of infants born to undernourished women, while for well-nourished women, there was no effect on birth weight [4]. However, it is well recognized that knowledge alone may not lead to behavior change. Several studies report multiple additional factors influencing nutrition practices during pregnancy, including healthcare providers’ knowledge, parents’ educational level, nutrition information provided, geographic location, beliefs and attitudes of mothers and the community, lack of family support, and poor access to foods [8–12].

Misconceptions, food taboos, and beliefs targeting pregnant women are reported as important factors affecting dietary practices [13–15]. Studies exploring nutrition knowledge and practices of pregnant women show that a diverse range of beliefs within indigenous knowledge systems is a powerful influencer of dietary practices [9–11, 16, 17]. In addition, certain foods may become associated with poor pregnancy outcomes leading to consumption of these foods being intentionally limited despite their nutritional value [5, 18]. These practices have been described in several African countries [14, 18, 19]. Also, some foods are restricted because of the perception that this will prevent the baby from becoming too big leading to difficulties during childbirth [5]. Such indigenous knowledge might be a barrier to the adoption of WHO recommendations for optimal dietary practices for pregnant women.

In the Democratic Republic of Congo (DRC), macro and micronutrients deficiencies remain a serious public health problem among women of reproductive age (chronic undernutrition 14%, anemia 39%) [20] despite efforts in nutrition education and community-based nutrition undertaken by Programme National de Nutrition (PRONANUT), under the Ministry of Health. Poor underlying maternal nutrition is a key risk factor for poor perinatal outcomes, including prematurity, low birth weight, and stillbirth. Although malnutrition in Kwango province, southwest of DRC, where this research was conducted, is particularly high [20–22], the provision of information regarding women’s feeding practices during pregnancy in this area is not available. To our knowledge, this is the first published research exploring dietary knowledge and practices, including pregnancy-related food taboos, among pregnant women in a rural context of the DRC.

## Methods

### Study site

The study was conducted in Popokabaka Heath Zone (HZ), one of the 516 HZs in the DRC. It is located in Kwango Province, which has many rivers and a tropical climate throughout the year. The population of Popokabaka is approximately 202 000 people. Popokabaka is isolated and highly inaccessible from developed areas, the closest urban area is over 10 h’s drive away, mainly on sand roads. The climate permits the cultivation of a wide variety of crops, as well as keeping livestock and fishing. Agriculture and farming are the most common activities in communities in the Kwango province. Women play an essential role in the cultivation of crops and food production. This is an area with high levels of poverty and unemployment, and malnutrition among pregnant women is prevalent. The most commonly spoken local language in Popokabaka is Yaka.

The study was conducted in two health areas of Popokabaka (*Popo City* and *Ingasi village*: 12 km apart from each other). The areas were purposively chosen to include both a peri-urban and a rural area in Popokabaka HZ.

### Study design and participants

A qualitative study was conducted in two health areas of Popokabaka HZ. The study population consisted of pregnant women, community members, and key informants. Key informants were health professionals involved in providing care to pregnant women and included facility health workers (Doctor, nurses, and nutritionist who provide antenatal care) and community health workers (CHW). All participants were purposely chosen based on their willingness to participate.

To be eligible, all participants have resided in the health area for at least 2 years. All pregnant women aged 18 years or above attending for antenatal care (ANC) were eligible to participate were approached to participate. Pregnant women were recruited in the ANC at the hospital and two health centers during the ANC. In the beginning, 12 pregnant women are targeted. Only nine consented, and completed to participate in the study. Older women were purposively selected based on having at least one grandchild, and husbands were selected based on being the father of at least one child, and were eligible to participate in focus group discussions (FGDs). Older women and husbands were not the family members of participating pregnant women.

Assisted by a community health worker (CHW), older women and husbands were recruited into the community and invited to participate in FGDs in the community one week before the interview. All participants approached consented and completed. All facility-based health workers and CHWs providing care for pregnant women were identified and invited to participate. Facility-based health workers were approached at health centers and CHWs were identified in the community.

### Data collection

Data were collected using in-depth interview (IDI) with pregnant women and health professionals, and FGDs with grandmothers and fathers. Qualitative data collection methods were chosen to explore in-depth information about the topic. FGDs were conducted separately among groups of grandmothers and fathers, this allowed for a dynamic interaction between the participants.

All IDIs and FGD were conducted using discussion guides and in the local language Lingala (Yaka) or French according to the preference of the participants. The guides covered topics including food recommendations during pregnancy, advantages of good nutrition during pregnancy, food habits, and food beliefs during pregnancy. To ensure privacy, interviews were conducted at the residence and the office with pregnant women and health professionals respectively. FGDs were conducted in each village at the nearby health centers. Data collection was stopped when saturation was reached. An experienced qualitative researcher assisted by a trained field worker, who was able to speak three local languages, collected all data.

### Data analysis

All interviews and FGDs were audio recorded using digital audio recorders and notes were taken by the field worker. The audios were transcribed verbatim by the team of three assistants by the investigator, and transcripts were translated into French and English. Transcripts were quality controlled by re-listening to the audio recordings and comparing with transcripts. Data were analyzed according to the inductive thematic approach. To increase coding validity, independent codes were created from a few interviews by two researchers and compared until a coding framework was agreed on. Finally, triangulation of IDI and FGDs was used to validate the data.

### Ethical considerations

The proposal received ethical approval from the ethics committees of the Kinshasa School of Public Health. Participation was voluntary. All participants provided written informed consent after a full explanation of the nature, purpose, and procedures used in the study. The participants were informed that responses will be anonymous and that they were free to withdraw from the interview or discussion at any time.

## Results

A total of nine IDIs were conducted with key informants; these were the doctor [1], the nutritionist [1], nurses [4], and CHWs [3] providing antenatal care. The IDIs were also conducted with pregnant women [9]. Four FGDs were conducted, two with husbands (one in each area) and two with grandmothers (one in each area) (Table 1). There were eight participants in each FGD. The results are reported for pregnant women, and key informants separately.

**Table 1:** Participants in IDIs and FGDs in each of the two areas

	Peri-urban	Rural	Total
*In-depth interviews (n* = *18)*			
Doctor	1		1
Nutritionist	1		1
Nurses	3	1	4
CHW	1	2	3
Pregnant woman	4	5	9
*Focus group discussions (n* = *4)*			
Husband	8	8	16
Older women	8	8	16

The mean age of pregnant women was 23.3(SD ± 7.6) years and most were housewives. The extended family was the main family type at both sites (Table 2).

**Table 2:** Sociodemographic characteristics of pregnant women (IDI: *n *= 9)

Variables	Popo city (*n* = 4)	Ingasi Village (*n* = 5)	Combined
Age in years (mean ± SD)	21.7 ± 8.0	24.6 ± 7,9	23.3 ± 7.6
Age of Pregnancy in weeks	30 ± 4	26 ± 3.4	27.7 ± 4.0
*Schooling of pregnant women*			
No schooling (*n*)	0	1	1
1–5 years (*n*)	0	1	1
6–12 years (*n*)	4	3	7
*Occupation (n)*			
Student	1	0	1
Housewife	3	2	5
Trader	0	1	1
Farmer	0	2	2
*Family type (n)*			
Nuclear	1	1	2
Extended	3	4	7
Household size (mean ± SD)	5.2 ± 1.2	5 ± 1.5	5.1 ± 1.3

### Pregnant women

#### Nutritional and diet knowledge

The level of knowledge among participating pregnant women showed a mixed picture with women showing good general knowledge about nutrition and the need for increased and more varied foods during pregnancy. However, they had little technical knowledge about nutrients and sources of nutrition. Healthcare workers (HCW) suggested that this might have been due to poor levels of education among women within the Popokabaka area.

During pregnancy, a woman must eat vegetables (amaranth), cassava leaves, meat….but according to her taste. She can take only vegetables with cassava bread or only fish. (Pregnant women 05, Popo City)

Protein: yes, in foods such as oranges, and amaranth. As for fats and carbohydrates: I've never heard of that. (Pregnant woman 01, Popo City)

#### Advantages of a good diet during pregnancy

The pregnant women had the same opinion about the benefit of a balanced diet during pregnancy. Good maternal health, good child development, prevention of anemia, and prematurity were the main advantages listed.

The first advantage, in my opinion, is the health of the child you carry in your womb and also the health of yourself as well. Wherefore you have to eat very well. The second advantage is that a pregnant woman provides food to the fetus via blood. Thus, she does not have to spend a day without eating. (Pregnant woman 02, Ingasi village)

#### Sources of information

The mothers reported that the ANCs, family, and community were the main sources of information for pregnant women. Therefore, family (grandmothers especially) play a determining role in the nutrition of the pregnant woman. Women who reported that they usually recommend foods to be eaten by a pregnant woman also considered older family members and older community members as a source of information. When a pregnant woman was asked where she get the information she said:

I learned them from the elders (dad, mom); but also some elders in the village. I also learned them in the maternity ward at ANCs where we are invited to eat all the foods that can give us weight. (Pregnant woman 02, Popo City)

I had got this information from my parents and some seniors in the village as well. I also got them at the ANC where we are invited to eat all the foods that can give us weight. (Pregnant woman 01, Ingasi village)

#### Dietary practices during pregnancy

The most consumed food items reported among pregnant women were cassava flour, cassava leaves, cowpea, and amaranths.

Here we eat hard. We usually eat cassava leaves, amaranths, sorrel, tomatoes; fish we do not eat frequently. Fufu made from cassava flour combined with maize flour is not frequently consumed. As for me, I eat fufu (usually made from cassava flour), cassava leaves, and amaranths. In addition, fish, goat meat, and cow meat. (Pregnant woman_08, Ingasi village)

Fruits were not commonly available in women’s diet, and among pregnant women who reported having eaten fruits, oranges were the most common fruits eaten. Some participants mentioned that they occasionally eat bananas, papaya, mangos, and avocado. Low fruit consumption was due to seasonality and lack of money to buy in the market.

Oranges, papaya. The oranges I used almost every day before the orange season ended. (Pregnant woman 01, Popo City)

In terms of food quantity consumed during pregnancy, pregnant women reported that they must be eating enough because they are obliged to ensure their well-being and especially that of their baby even if health providers and community members considered that the quantity consumed by pregnant women was not enough. Therefore, food intake varied among women. They would eat once, twice, or three times a day; only one woman reported eating snacks in between her meals.

I usually eat 3 times: in the morning I have tea and bread; at noon I still take it and in the early evening it's the big meal. (Pregnant woman 04, Popo City)

A pregnant woman should eat more; this is the case for example with my mother when the meal is in insufficient quantity, she prefers first that I eat and she will eat after. She always told me a pregnant woman should eat more. In addition, at the ANC, I was told the same thing. (Pregnant woman 01, Ingasi village)

#### Key informants (older women, husbands, CHWs, nutritionist, and doctor)

##### Perceptions of mothers’ nutritional knowledge

Fathers and grandmothers perceived that pregnant women have poor knowledge about nutrition and dietary practices. They reported that because of a lack of knowledge women did not follow the recommended dietary intake such as frequently eating at least three times a day, and increasing the variety and quantity of food. To try to close this gap, HCWs informed mothers about nutritious food and balanced dietary intake during nutritional education when attending ANC.

There are women who know and others do not. There are many who don't know. There are moms who hardly eat. (Father 02_Ingasi village)

I think they don't have enough knowledge because they eat anything and anyway. (Grandmother 02, Popo City)

Some know; they learn something from our teachings. Others do not, given their level of education. (HCW 03, senior nurse)

##### Key informants' nutritional knowledge

In contrast to the pregnant women themselves, both fathers and grandmothers appeared to be knowledgeable about a healthy and balanced diet. They were able to identify the sources of food and describe a balanced diet. They reported that a pregnant woman must consume everything, especially vegetables, cereals, legumes, fish, meats, tubers, and fruits as well. The food quantity, the quality, the variability, and the frequency of meals should be ensured.

A pregnant woman has to eat 3 times, morning-noon-night, or even more. And not just cassava flour; it must vary, also taking peanuts, plantains, yamies. (Grandmother, Popo city)

##### Sources of mothers’ foods consumed

Having a balanced diet requires an individual to have different sources of food and means of acquiring food. The community of Popokabaka accessed food through farming, fishing, livestock, and in the market. The most common source of food production within the community was farming. Since poverty is prevalent within the Popokabaka area participants invested more in growing crops, livestock, and fishing. Some of the harvested food would be taken to the market for selling, and livestock is commonly used to perform traditional ceremonies. As stated by the nutritionist below, farming, market, and livestock were the source of food but small livestock was not for household consumption because they had a traditional or social function:

Food comes from both the fields (local products) and the market. But the predominance is more of local products. As local products, we have cassava, sesame, cowpea, corn, goat, and poultry. For cowpea, there is also some restriction, especially in the last trimester of pregnancy that is, from the 6-7th month. The woman runs the risk of bleeding a lot during childbirth. Goat meat is not often eaten because small livestock farming is intended to solve social problems that may arise such as school fees. (HCW 02, nutritionist)

Pregnant women also accessed food from the market. In the market, there was a variety of food, which could improve dietary diversity leading to a more balanced diet. However, participants were limited by the lack of money to buy their desired and alternative food.

Food comes from the fields much more but also from the market. But often you don't have the money to buy. (Father 01, Ingasi village)

##### Sources of mothers’ nutritional information

According to key informants, nutritional information was available from healthcare facilities, media, NGOs, and family members. In healthcare facilities, the main informants were HCWs and community healthcare workers (CHW). During ANC, visits by HCWs such as the nutritionist and nurses would teach mothers about food consumption and nutrition. CHWs would visit women in their homes and give them advice relating to optimal eating practices during pregnancy.

I do it very often. I usually organize home visits. I tell them that you must always eat; never work starved. Before going to the fields, you have to eat. Eat in the morning, at noon if you do not go to the fields, and in the evening. If you stay hungry the child you are carrying remains as hungry. (CHW01, nurse)

Participants mentioned several nutritional education programs that have been implemented by Non-governmental organizations (NGOs) within the village to address malnutrition challenges among women and children.

There was the intervention of the NGO [ACF] who told pregnant women that they are supposed to eat everything so that they are healthy. There is also another project funded by the NGO. (Father 01, Popo city)

##### Perceptions of mothers' dietary practices

In terms of quantity, all key informants knew that a pregnant woman must have enough food on her plate, unlike other ordinary people, to ensure the well-being of both the mother and the child. One of the fathers reported that pregnant women do not eat as required because they often skip meals when doing their chores such as collecting woods in the forest. In times when the food is not sufficient, some family members would give priority to a pregnant woman to eat first.

In the village, they don't know. This is why they often go out in the morning, they go to the forest without eating until the evening. (Father 04, Ingasi village)

They do not know because when it comes to eating, they give more to the husband and the children. Other pregnant women are those we call 'SAFRICAS', that is the women who work all day long forgetting even that they must eat for their well-being and their baby. (Older woman 02, Ingasi village)

Although women knew that they should eat the right quantity of food and eat more frequently, health care workers (HCW) felt that that women's diet did not change when they became pregnant; they continue to eat the same meals mentioned below.

‘Generally, they are used to eating amaranths, cassava leaves, cowpea, sesame, fish if available, FUFU. (HCW03, senior nurse)

The HCW also believes that the nutrition practice is low in fat and protein. They rarely consume fruits. One health worker said:

Pregnant women rarely eat amaranths and fruits. As far as meat is concerned, it is even rarer. They prefer eating Mbondi (a kind of amaranth) without even mixing it with the oil. So the quality is not good. (HCW02)

##### Barriers to optimal dietary practices during pregnancy

Poverty within the Popokabaka area was a big challenge for pregnant women to improve their nutrition. Although not all participants mentioned they were living in poverty, it was clear that their living conditions were poor and that households had a low income and limited resources for food production, lacked food variety, and a few women had a low level of education. One of the HCWs stated that it is difficult for them to change dietary practices among pregnant women because of poverty.

It is difficult. Given the environment and poverty from a food point of view, it is difficult for them to have a good diet. (HCW05, senior nurse)

Lack of knowledge among some pregnant women has resulted in them not paying attention to the essential healthy behaviors during pregnancy. Fathers reported that women have a poor understanding of the importance of attending ANC and other necessary sources of information provided to them, and they are influenced by traditional customs; hence, they have limited food knowledge.

Some go to the NPC [ANC], thanks to their husbands' knowledge of the importance of NPC [ANC]; others, on the other hand, are not because of their stubbornness or their ignorance, or the influence of custom (father 08, City)

Because of lack of education, information, and lack of money. (Father 08, Ingasi village)

They do not understand and do not have this knowledge, and it is the medical training that must teach them what to eat. (Father 01, City)

HCWs agreed that women lack information, but when they provide nutritional information to women, they are often unable to comply as a result of negative influence from family members, including the father of the child, who dismiss the information provided to pregnant women.

First, it is the lack of information, especially for women from the villages near the city. Also, the food bans [taboos] that are numerous. Also, other pregnant women eat anyhow. For example, tea, pregnant women should take it moderately but excess tea can be a poison for the child. This is also the case with alcoholic beverages called Lotoko (corn-based alcohol) that some pregnant women take regardless of the dose. There is also the influence of the pregnant woman's family especially husbands and elderly mothers. They tend to boycott everything that health care workers teach women at ANCs. There is also the level of education because most of our women here have not studied. That is why we always have to insist and raise awareness regularly to get them to internalize this. (HCW 02, Nutritionist)

Furthermore, pregnant women tend to attend ANC at the later stage of their pregnancy, mostly between six to seven months of pregnancy. Late ANC attendance becomes a barrier for HCW whose job is to guide and supervise women toward a healthy diet and pregnancy.

There is also a mutual influence between them especially on the beginning of ANC: [other mothers say] why do you start the A NC so early? You are only the third month; wait until the sixth or seventh to get there. (HCW 05, senior nurse)

The family played a significant role in women’s pregnancy life. They support and guide them throughout the pregnancy, particularly the baby’s father and older women. HCWs reported that family members had strong opinions, which were not always constructive. There were foods that a pregnant woman was told she should not eat or should eat in small quantities due to the perception that the baby will be big. Such information was mostly coming from family members.

From the 6th month, she cannot eat cowpea, sesame for fear that the child is too fat. (Father 03, Ingasi village)

Although food quantity and variety were a priority for pregnant women, certain foods were prohibited from being eaten, mainly during the last trimester, due to food taboos that society has concerning pregnant women. Food taboos were a huge barrier to improving nutrition among pregnant women in the village, considering that they had limited food options. Such attitudes were concerning to HCWs, who believed that mothers do not implement what they were taught during ANC due to societal influence.

As far as the practices of pregnant women in our community are concerned, it is really difficult because they have a lot of prohibitions, especially concerning animal proteins and eggs as well. For animal proteins, such as pork, pangolin meat, and civet. They do not consume pork because they think that after birth, the child will develop pig-like cries; for the pangolin, it will develop a cough. For eggs, the woman will give birth to a child without hair. (CHW02_Nutritionist)

Food taboos existed commonly in foods that were nutritious and important for pregnant women to eat. These foods include pork, eggs, chicken, cowpea, certain fish species, goat meat, mushroom, and many others. The perceived reasons for not consuming such foods are as follows:

- Pork meat causes pork-like cries in children
- Eggs cause the absence of hair in children
- Sorrel leaves lead to the deficiency of breast milk
- Mushrooms increase the risk of malnutrition in children
- Caterpillars are responsible for incessant crying in children
- Duck meat causes yellow fever
- Chicken causes epilepsy in children
- Red fruits and vegetables-they believe that the baby will have a red anus

However, food taboos were not consistently practiced throughout the community in Popokabaka but varied according to family traditions.

There are more related to family prohibitions. Some pregnant women should not, for example, take eggs or goat meat (causing muscular weakness during childbirth) or duck meat, or pork. Others also chicken causing epilepsy in children, guinea fowl; it depends on the doctrine of each family. However, it is not all pregnant women who are affected. It depends on the doctrine of one family to another. They don't want to eat the foods that can build their bodies. (CHW 01)

## Discussion

Our study shows that women in this rural community in DRC had a good basic knowledge of the main messages relating to diet during pregnancy, having received information from the antenatal clinic, community health workers, and community nutrition projects. However, they were unable to apply this knowledge in practice as a result of a combination of social, cultural, and economic factors. Our findings describe pregnant women who, as a result of extreme poverty, had little or no access to the variety of foods sold in the market, agriculture was the primary source of food production, and women relied mainly on the foods they grow. As a result, most women have a monotonous, largely plant-based diet. Pregnant women frequently worked long hours in the fields and the home, sometimes missing meals as a result. In addition to poverty, pregnant women’s diet was strongly influenced by family members, cultural food taboos, gender roles in the household, and incorrect perceptions that weight gain during pregnancy would lead to a difficult birth. All of these factors contributed to women lacking the variety and quantity of foods in their diet required for a healthy pregnancy, putting their health and the health of the baby at risk.

Poverty and food insecurity are important determinants of malnutrition, and malnutrition remains unacceptably high among pregnant women in many parts of sub-Saharan Africa, including DRC [23]. Pre-existing malnutrition and poor diet in pregnancy are associated with poor maternal and neonatal outcomes, and maternal malnutrition can have lifelong effects on the health of the child [24]. Since the communities in Popokabaka rely mainly on their crops, an important intervention to improve nutrition and dietary diversity would be to capitalize on the agricultural sector. A wide variety of approaches to improve agricultural practices has been shown to decrease poverty, and improve food security and food diversity in rural Africa. Effective interventions could include smallholder farmer training, irrigation infrastructure rehabilitation, provision of drought-resistant seeds and fertilizer, rainwater harvesting, improving food storage, planting of backyard gardens, and introduction of loan schemes to support smallholder farmers. [25, 26]. For example, the implementation of a backyard garden project within the Ramotswa area in Botswana was able to successfully reduce poverty in participating households from 52 to 15% [27]. Similar practices could be adopted and evaluated to strengthen agricultural practices in the Popokabaka area, improve dietary diversity and alleviate poverty. However, such interventions require technical support, guidance, and supervision to be successful. NGOs in the local area could be mobilized to provide this support. Research is needed to find feasible, context-specific, and effective approaches to enhance productivity and increase the range of crops grown in a particular environment. Research should further demonstrate that such interventions could effectively improve pregnant women’s diets.

Although poverty is a leading cause of malnutrition in pregnant women, cultural beliefs and practices during pregnancy also play an important role. Food taboos, defined as the restriction of particular foods due to culture, religion, or perceived health benefits, are common in all populations, including in developing countries [28]. In particular, food taboos are commonly practiced by pregnant women [14, 18, 29]. In our study, community members described a wide variety of food restrictions for pregnant women including many sources of protein like eggs, meat, types of fish, caterpillars, as well as some vegetables and mushrooms. Food restrictions are intended to protect pregnant women and their unborn babies from adverse outcomes perceived by the community to be associated with eating particular foods [14]. In our study, local foods were described as causing a wide variety of conditions in the baby from lack of hair to malnutrition and epilepsy. However, these restrictions are not aligned with medical recommendations for optimal diet in pregnancy, and they reduce pregnant women’s access to important sources of protein and other nutrients, particularly in a setting like rural DRC, where dietary diversity is poor. Food taboos made it more difficult for pregnant women in our study to have a healthy balanced diet and this was likely to lead to adverse pregnancy outcomes. Societal structures such as traditional leaders, religious leaders, and family members ensured that information about food taboos was passed intergenerationally, and older women and husbands supervised pregnant women to ensure that they do not consume foods that are culturally prohibited. Studies in a variety of settings suggest that pregnant women adhere to such restrictions despite the advice given by health workers [14].

Gender norms and values also play an important role in determining women’s dietary practices and their ability to comply with dietary recommendations. This is shown in many settings where women frequently take disproportionate responsibility for household chores and childcare, and are expected to put other family members’ nutrition needs before their own. In our study, pregnant women lacked autonomy and were obedient to cultural practices, including complying with food taboos. In addition, family gender norms meant that pregnant women reported performing strenuous daily chores such as fetching water and firewood or walking long distances to work in the fields, sometimes missing meals. In many settings, food access and distribution within the household is gender-based, for example, a woman prepares a meal, but she must first dish for her husband, then the children, and lastly herself, irrespective of her pregnancy or nutritional needs [18]. In a study by Rosen et al., in Niger [5], women stated that their diet was restricted by their husbands who hold authority over food acquisition, making it impossible for women to improve their dietary practices. Thus, malnutrition among women in developing countries is exacerbated by social norms and gender-based discrimination which require women to put family members before their health and nutritional needs [30]. We suggest that women need to be liberated from cultural and gender norms, which control their well-being.

Research has shown that education and financial freedom gives women autonomy [5], and this is supported by the findings by Oni & Tukur (2012) which show that women who are educated and have their income can resist cultural influences on their dietary practices. Whereas younger women with low education and pre-existing malnutrition were more likely to adhere to food taboos [31]. Therefore, investing in women’s empowerment might improve nutrition among women and children. However, long-term cultural practices are resistant to change, and achieving change requires a long-term investment in female education and the provision of opportunities for income generation and employment for women. This may be very difficult to achieve in a deep rural area like Popokabaka. However, it may be possible for interventions to improve agricultural practices to be linked to income generation schemes for women that could contribute to women’s empowerment in the area.

Regarding the conflict between cultural views and the health system, the justification for food restrictions is to protect the health of the pregnant woman and her baby, but in many cases, food taboos are directly in opposition to the advice given to mothers by HCWs. In this study, HCWs reported that family members disregarded information given to pregnant women, and this has been shown in other studies [14, 31, 32]. Mothers and the broader community need to understand the potential adverse health impacts that may result from a poor diet, including stillbirths, premature birth, low birth weight, and maternal and neonatal death [33]. However, it is also clear that understanding the cultural context of food consumed during pregnancy is important for all messaging about nutrition for pregnant women. There is a need to provide health education to pregnant women that is acceptable for the mother, her family, and the community, and that messages should be tailored to comply with local food taboos wherever possible. The health system should play a strong role in educating, not only the mother but also the community at large, about the importance of nutrition for pregnant women and the dangers of restricting nutritious foods during pregnancy. There are resources in the community, including facility-based HCWs, CHWs, and NGOs with an interest in nutrition projects, who can work together to address the concerns highlighted by this research. Approaches could include community nutrition days, community mobilization to address malnutrition, and encouraging attendance of family members at ANC. Further, mothers with a low Body Mass Index (BMI) pre-pregnancy are at a much higher risk of adverse outcomes [34], and these mothers should be targeted for additional support during their pregnancy to ensure adequate nutrition and weight gain.

## Conclusion

Poor dietary practices among pregnant women in rural DRC are linked to poverty, food taboos, and gender norms, and put women and children at risk of malnutrition and lifelong adverse outcomes. A multi-pronged approach is needed to address this challenge, which should include improvements to agricultural practices, improved education for women and the community, as well as women’s empowerment. Further research is required to evaluate interventions so that effective interventions can be rolled out to other vulnerable communities.

## Data Availability

All data, transcripts, and supporting documents for this study are available at the Kinshasa School of Public Health, University of Kinshasa. They will be made available upon request to the leading author.

## Abbreviations

NORAD: Norwegian Agency of Development
DRC: Democratic Republic of Congo
GROWNUT: Growing Partnership for Higher Education and Research in Nutritional Epidemiology
FGD: Focus group discussion
IDI: In-depth interviews
ANC: Antenatal care
HZ: Health zone
HCWs: Health care workers
CHWs: Community health workers
NGO: Non-governmental organization
BMI: Body mass index
